# Integrative neurobiology of metabolic diseases, neuroinflammation, and neurodegeneration

**DOI:** 10.3389/fnins.2015.00173

**Published:** 2015-05-18

**Authors:** Gertjan van Dijk, Steffen van Heijningen, Aaffien C. Reijne, Csaba Nyakas, Eddy A. van der Zee, Ulrich L. M. Eisel

**Affiliations:** ^1^Department Behavioural Neuroscience, Groningen Institute for Evolutionary Life Sciences, University of GroningenGroningen, Netherlands; ^2^Systems Biology Centre for Energy Metabolism and Ageing, University Medical Center, University of GroningenGroningen, Netherlands; ^3^Department Molecular Neurobiology, Groningen Institute for Evolutionary Life Sciences, University of GroningenGroningen, Netherlands; ^4^University Centre of Psychiatry, University Medical Center Groningen, University of GroningenGroningen, Netherlands

**Keywords:** neuroinflammation, obesity, metabolic syndrome, type-2 diabetes mellitus, TNF, blood-brain barrier, aging, Alzheimer's disease

## Abstract

Alzheimer's disease (AD) is a complex, multifactorial disease with a number of leading mechanisms, including neuroinflammation, processing of amyloid precursor protein (APP) to amyloid β peptide, tau protein hyperphosphorylation, relocalization, and deposition. These mechanisms are propagated by obesity, the metabolic syndrome and type-2 diabetes mellitus. Stress, sedentariness, dietary overconsumption of saturated fat and refined sugars, and circadian derangements/disturbed sleep contribute to obesity and related metabolic diseases, but also accelerate age-related damage and senescence that all feed the risk of developing AD too. The complex and interacting mechanisms are not yet completely understood and will require further analysis. Instead of investigating AD as a mono- or oligocausal disease we should address the disease by understanding the multiple underlying mechanisms and how these interact. Future research therefore might concentrate on integrating these by “systems biology” approaches, but also to regard them from an evolutionary medicine point of view. The current review addresses several of these interacting mechanisms in animal models and compares them with clinical data giving an overview about our current knowledge and puts them into an integrated framework.

## Background

Over the last century, the life span of humans living in technologically advanced societies has expanded dramatically due to the vast knowledge gained about mechanisms underlying sustainable health and disease, and how to implement this knowledge in the health care system. As a result, a higher percentage of the population in these societies become centenarians as ever before, yet certain life-style related diseases remain a problem as well. In fact, it is even questionable whether the population as a whole is aging more healthily than before. Major obstacles to sustainable health are metabolic diseases including obesity and diabetes mellitus (World Health Organization, 2015a,b). These diseases appear to be attracted quite easily, yet they are very difficult to treat, and can have a devastating impact on sustainable health of individuals on the long-term (World Health Organization, 2015a,b). It is becoming increasingly clear that diabetic and obese individuals have an increased risk to develop neurodegenerative diseases on the long-term (Craft, 2005; Profenno et al., 2010). Because the incidence of infant obesity and diabetes mellitus is on the rise, it is expected that brain derangements like Alzheimer's disease (AD) will increase in the future too. AD prevalence is estimated to reach 106.2 million people worldwide in the year 2050 (Brookmeyer et al., 2007). If one were able to delay the onset of this disease with 12 months, this would yield 9.2 million less cases of AD worldwide (Brookmeyer et al., 2007). A common characteristic of metabolic diseases is a chronic and low-grade activation of the inflammatory system (Gustafson, 2010; Vykoukal and Davies, 2011). This inflammation may eventually spread from peripheral tissue to the brain, and recent evidence indeed suggests that neuroinflammation is an important causal mechanism in AD (Hanzel et al., 2014). The current review aims at providing an integrative neurobiological framework of the processes that culminate from behavioral, metabolic, endocrine, and energy balance derangements into neuroinflammation and neurodegeneration.

## Inflammation and metabolic diseases

Inflammation is a necessary and evolutionary conserved response to harmful stimuli that can include tissue damage or various pathogens that invade the host. The inflammatory response includes numerous mechanisms and cell types (i.e., platelets, neutrophils, macrophages, fibroblasts, endothelial cells, nerve cells, and lymphocytes) interacting with each other to clear out the causes of the injury, limiting ongoing infection and/or compartmentalize damaged tissue, and to initiate tissue repair aimed at restoring normal physiological function (Nathan, 2002). Among others, Toll-like receptors (TLRs) on macrophages are involved in pathogenic pattern recognition to generate immunologically relevant responses (Takeuchi and Akira, 2010). The release of the cytokine Tumor Necrosis Factor-alpha (TNFα) is the prototypical response underlying inflammation and makes up the acute phase reaction (Kmiec, 2001). Locally, TNFα promotes cell death of damaged tissue through an apoptotic caspase-dependent pathway (Rath and Aggarwal, 1999) allowing repair of cells and tissue worth repairing (Winston et al., 1999). However, TNFα also stimulates a cell survival pathway through the nuclear factor kappa B (NF-κB) (Gupta et al., 2005). Locally produced insulin-like growth hormone (IGF-1 and IGF-II) orchestrates different biochemical events that culminate into the restoration of functional integrity of damaged tissue (Bitar, 2000). Systemically, Growth Hormone (GH) released from the anterior pituitary influences this repair process too by stimulating the production of IGF from the liver (Dunaiski and Belford, 2002). IGF stimulates, for example, glucose uptake into injured tissue (Rajpathak et al., 2009) thereby promoting energy availability for tissue repair (Singh et al., 1998). Inflammatory cytokines also stimulate the liver to secrete triglyceride-rich lipoproteins, which can be used to fuel the host's response to infection, clear pathogens and bacteria, and may exert immune-modulatory effects that help the innate immune response to inflammation (Harris et al., 2000; Barcia and Harris, 2005). Glucocorticoids acting through glucocorticoid receptors (GR) efficiently suppress the micro-environmental IGF system (Bitar, 2000) and suppress cytokine induction downstream of TLRs (Ogawa et al., 2005), presumably involving mitogen-activated protein kinases (MAPK) activation (Bhattacharyya et al., 2007). Finally, glucocorticoids reduce GH release (Tulipano et al., 2007) through a hypothalamic somatostatin-dependent pathway. GR belongs to the nuclear-receptor superfamily of transcription factors, to which also the liver-retinoid receptor (LXR) and peroxisome-proliferator-activated receptors (PPARs) belong, and these have emerged as important modulators of innate immunity through their effects on macrophage gene expression (Castrillo and Tontonoz, 2004).

When the compensatory response (inflammation) is not able to repair the damaged tissue, for example due to chronically increased GR activation, this will turn into a chronic condition, with continuously heightened levels of triglycerides and cholesterol, which can lead to the formation of atherosclerotic lesions (Esteve et al., 2005). Conditions that could easily underlie these types of inflammations are commonly found in obesity, and this may increase the risk for metabolic disease like type-2 diabetes mellitus (Dandona et al., 2004; Vykoukal and Davies, 2011; Grant and Dixit, 2015).

### Metabolic diseases

Although obesity is simply the consequence of energy intake exceeding energy expenditure, the underlying mechanisms are multifactorial. First of all, energy intake and expenditure are linked with body fat as a regulated factor. This adipocentric hypothesis–originally coined by Kennedy in the 50s of the previous century (Kennedy, 1953)–is now well-established and was reinforced by the discovery of the adipocyte hormone leptin by Zhang et al. (1994). Brainstem and hypothalamic neuropeptidergic neurocircuitry down-stream from, among others, leptin, are involved in the homeostatic control of food intake and energy balance, and alterations in these pathways most often lead to secondary leptin resistance (Schwartz et al., 2000), and often underlie weight gain (Gao and Horvath, 2008; Zeltser et al., 2012). On top of this homeostatic control mechanism is the mesolimbic neurocircuitry involved in hedonic aspects of food (Berridge, 2009; Berthoud, 2011). These reward pathways can also be inhibited by leptin, thereby dampening food-associated reward (Farooqi et al., 2007; Davis et al., 2010). There are several other ways that affect the trajectory over which obesity establishes itself (McAllister et al., 2009), however an increase in the accessibility of highly palatable food items with a highly saturated fat content and refined sugars is a leading cause of weight gain in obesity-prone individuals, as such a diet easily overrides homeostatic control mechanisms of energy balance (Berthoud, 2011). At this point, it is important to realize that these mechanisms have been shaped by evolutionary processes, favoring genes and traits for ingestive behavior and weight gain in times of plenty allowing survival in time of famine (Dallman et al., 1993; Chakravarthy and Booth, 2004). In modern industrialized societies famine is obviously not an issue anymore, yet these genes dealing with it are probably rooted deep in our genetic architecture (Chakravarthy and Booth, 2004), although other theories also exist (Speakman, 2006). For these reasons, achieving successful weight loss is currently extremely difficult. Eating highly palatable food can easily become addictive, and abstinence from such food items can activate withdrawal mechanisms (Avena, 2007; Hoebel et al., 2009). Temporary reductions in energy intake leading to weight loss can trigger sustainable reductions in energy expenditure that oppose further weight loss (Rosenbaum and Leibel, 2014), and these changes can persist long after relapse toward derailed eating behavior of palatable and usually energy-dense food.

At some point in the progression of weight gain, adipose tissue attracts monocytes and macrophages and changes its endocrine functioning (Permana et al., 2006). While the exact mechanisms are still subject of intense research (Ye, 2009; Goossens and Blaak, 2012), it is known that adipocyte/macrophage interactions lead to production of cytokines and induce local as well as systemic inflammatory mechanisms (Fain, 2006; Wood et al., 2009). TNFα is known to be elevated in obese individuals, particular in those with visceral adiposity (Tsigos et al., 1999). TNFα causes insulin resistance by direct inhibition of insulin receptor tyrosine kinase activity (Hotamisligil et al., 1995). In addition, IgG antibodies linked to adipose B cell accumulation may play a role in the development of insulin resistance in target tissue as well, since this is attenuated by B cell depletion and conversely augmented in lean mice that underwent IgG transfer from diet-induced obese mice (Winer et al., 2011).

TNFα induces the secretion of other proinflammatory cytokines, such as interleukin (IL)1β and IL6, and reduces the secretion of the adipokine adiponectin (Wang and Trayhurn, 2006). Adiponectin increases fat oxidation in peripheral tissue, and promotes mitochondrial biogenesis, and reduces inflammatory markers (Yamauchi et al., 2003; Greenberg and Obin, 2006; Lionetti et al., 2009). A reduction in plasma adiponectin levels, at least of its high molecular weight form, has been associated with the development of metabolic syndrome and type-2 diabetes mellitus irrespective of changes in adipose tissue mass (Yamauchi et al., 2001; Hojlund et al., 2006). A comparable inhibitory effect of TNFα on the anti-inflammatory and extracellular matrix stimulator Transforming Growth Factor [TGF]-β has been observed (Yamane et al., 2003). The anti-inflammatory factor IL10 conversely inhibits the TNFα system by inhibiting TNFα converting enzyme (TACE) in monocytes (Brennan et al., 2008) and IL10 reduces the TNFα-induced NF-κB pathway activation as well as the apoptotic mechanisms (Dhingra et al., 2009). Although IL10 receptors seem a promising target for treatment of metabolic/inflammatory diseases, however conflicting results have been found in which IL10 in fact accelerates pancreatic Beta-cell failure (Balasa et al., 2000).

TNFα promotes spill-over of fatty acids from visceral adipose tissue toward the liver, which contributes to elevated hepatic triglyceride (TG) production and reduced Very Low Density Lipoprotein clearance (Feingold et al., 1990). Consistent with this is the finding that TNFα gene knockout mice have increased visceral fat deposition, but have far lower hepatic fat storage than wild-type mice (Salles et al., 2012). While the classical lipid changes associated with the metabolic syndrome (i.e., increased triglycerides and lipoproteins) may be envisioned as a highly conserved evolutionary response aimed at tissue repair (Esteve et al., 2005), these metabolic and inflammatory mechanisms can become maladaptive and underlie the etiology of cardiovascular and metabolic diseases (Popa et al., 2007).

### Central nervous control of substrate homeostasis

In the progression from weight gain to inflammation the autonomic nervous system appears to play a major role. Obesity is frequently associated with a decline in parasympathetic activity and (regionally) increased sympathetic activity (Arrone et al., 1997; Vaz et al., 1997; Grassi et al., 2005; Skrapari et al., 2007; de Jonge et al., 2010). Increased sympathetic activity is already observed before obesity establishes itself, since diet-induced obesity prone rats can be characterized before access to a palatable high fat diet by the hyper-responsiveness of the sympathetic nervous system (Levin, 1993). Increased sympathetic activity and increased blood flow through adipose depots causes the release of fatty acids which either can be used for metabolic purposes, or can be re-esterified in fat but also in extra-adipose tissues including the liver and muscle (Bjorntorp, 1991). Free fatty acid (FFA) spill-over from visceral fat into the portal circulation is caused by increased sympathetic activity in the visceral abdominal depots, and this can easily become a self-perpetuating process because increased FFA levels, particularly short-chain, can activate the sympathetic limb of the autonomic nervous system as well as the hypothalamo-pituitary-adrenal (HPA) axis (Benthem et al., 2000). A diet with a high level of saturated or mono-unsaturated dietary fat contributes far stronger to abdominal weight than a diet with a poly-unsaturated fatty acid (PUFA) content (Jang et al., 2003). Eventually, this redistribution of fat from adipose to extra-adipose stores can worsen insulin resistance and lead to gradual organ failure (Unger and Scherer, 2010), provided that food intake is not obstructed (Unger and Scherer, 2010). Whether local intra-abdominal secretion of pro-inflammatory cytokines is increase as a consequence of sympathetic outflow in obesity needs to be established. This possibility may be plausible since TNFα secretion in visceral adipose tissue is increased by sympathetic activity induced by ischemic stroke (Wang et al., 2011). Reduced parasympathetic activity, perhaps as a result of a shift in the autonomic balance, is an unfavorable condition as well since the efferent vagus nerve by means of cholinergic stimulation–is necessary to balance cytokine production from macrophages (Tracey, 2007).

Brain mechanisms underlying changes in autonomic outflow in obese subjects are yet to be discovered, however the hypothalamus may be playing a major role since it contains preganglionic motor circuits that regulate autonomic outflow (Luiten et al., 1985). These systems are sensitive to peripheral hormones including insulin and leptin and can regulate peripheral fuel fluxes (Marino et al., 2011). In addition, enhanced afferent reflex mechanisms from adipose tissue to the hypothalamus in obesity prone animals may contribute to increase sympathetic activity as well (Xiong et al., 2012). Because parts of the hypothalamus have a relatively leaky blood brain barrier (BBB) epithelium, it is no surprise that activated microglia (i.e., which are the innate immune cells in the CNS) and increased levels of IgG (Yi et al., 2012) are found in the arcuate nucleus (ARC) of the hypothalamus of mice subjected to a high fat diet (Thaler et al., 2012; Yi et al., 2012). Analogous to the periphery, the innate immune cells (microglia in the case of the CNS) are important for maintaining organ physiology and relieving cytotoxicity by clearing cell debris by phagocytotic mechanisms (Neumann et al., 2009). In this respect the increased levels of leptin and insulin, and several other peripheral hormones found to be elevated in obese individuals, have neuroprotective properties in the CNS (Signore et al., 2008). Also analogous to the periphery is the increased secretion in the CNS of inflammatory cytokines including TNFα as a result of feeding a high fat diet and/or the resultant diet-induced obesity (Thaler et al., 2012). It seems likely that increased TNFα expression in astrocytes is more relevant for cerebral pathology than TNFα expressed in neurons (Akassoglou et al., 1997). Increased TNFα can underlie hypothalamic insulin and leptin resistance through the protein tyrosine phosphatase—PTP1B pathway, which dephosphorylates the insulin and leptin receptor associated Janus kinase (Picardi et al., 2010). Hypothalamic insulin and leptin resistance in turn contribute to derangements in ingestive behavior, autonomic outflow and fuel fluxes, and eventually will augment the inflammatory and metabolic processes mentioned in the previous section that fuel the vicious cycle underlying the cardio-metabolic syndrome (De Souza et al., 2005; Posey et al., 2009; Thaler and Schwartz, 2010; Lumeng and Saltiel, 2011).

### Blood brain barrier integrity

Hormonal factors like insulin and leptin not only gain access to the hypothalamus (or other “leaky” regions like the area postrema and subfornical organ), but virtually all brain circuits are reached via receptor mediated transport located in the epithelium of the BBB (Banks et al., 1996). Because plasma leptin levels increase with higher levels of body fat (Considine et al., 1996), the level of leptin penetrating the brain would be expected to increase as well. This, however, is not the case since the transport system becomes impaired by circulating triglycerides associated with obesity and insulin resistance (Banks et al., 2004). Such a fat-induced blockade of leptin transport across the BBB seems maladaptive from an obesity-point of view (which in fact would be worsened by this mechanism). Starvation, however, is also characterized by hyperlipidemia, at least in the blood stream (Banks et al., 2004), and the ability of triglycerides to induce leptin resistance during famine counter the leptin-induced shift toward use of triglycerides as an energy source and so helps to conserve fat stores. With respect to insulin, obesity is also associated with reduced transport across the BBB (Baskin et al., 1985; Kaiyala et al., 2000), but this is not blocked but rather reversed by triglycerides (Urayama and Banks, 2008). Thus, in the case of obesity and insulin resistance, the levels of insulin penetrating the brain can be increased, provided high levels of circulating triglycerides.

Although the brain has a very high concentration of lipids, it is not necessarily dependent on fatty acid oxidation as a source of energy. Glucose is the most important energy-yielding substrate in the CNS, which is brought across the BBB endothelium predominantly by GLUT 1 and GLUT 3 transporters independent of insulin (Seaquist et al., 2001). Neurons then take up glucose directly (Patel et al., 2014), although a glucose-lactate shuttle has also been proposed in which astrocyte nourish neurons by lactate converted from glucose (Magistretti and Pellerin, 1999). Glucose transport in the cortex, cerebellum, hippocampus, and certain regions in the hypothalamus is dependent upon insulin, via GLUT4 and GLUT8. As with the hypothalamus, insulin resistance in these brain regions may be proposed to contribute to hepatic insulin resistance leading to glucose intolerance and hyperinsulinemia. As such, peripheral insulin resistance would be adaptive in order to counter-regulate diminished glucose availability for neuronal processes (McEwen and Reagan, 2004; Winocur et al., 2005). Consistent with this is the idea that glucose utilization is impaired in extremely obese Zucker compared to lean Zucker rats (Doyle et al., 1993). The Zucker rat is a widely used rat model for type 2 DM and is known to have impairments in hippocampal functioning that are related to reduction in hippocampal membrane associated GLUT4 and insulin receptor expression (Winocur et al., 2005). In addition, insulin dysfunctioning due to experimental (Malone et al., 2008) or clinical (Kooistra et al., 2013; Koekkoek et al., 2014) diabetes mellitus causes deterioration in cognitive functioning and higher risk for stress-induced damage (Grillo et al., 2003).

Recently, Davidson and colleagues observed that hippocampal BBB permeability of rats becomes increased (using a sodium fluorescein penetration test) when these animals were fed a high fat high sugar diet provided that they gained weight (Davidson et al., 2012). Rats that did not gain weight on this diet did not show increased leakage. The increased permeability was found to be associated with memory impairments, in particular hippocampus dependent learning (Davidson et al., 2012). Obviously, a leaky BBB exposes the brain to “secondary” risk factors that easily can do harm, and it is plausible that this underlies the obesity-associated inflammatory process, as was discussed previously. Monocyte/macrophage of which the levels are found to be elevated in obesity could be transported across the BBB and may facilitate this process, although this idea needs to be confirmed (Freeman et al., 2014). Associations between cognitive impairment and obesity are also found in the human literature (Fitzpatrick et al., 2013; Vainik et al., 2013). Causal mechanisms by which obesity affects BBB integrity are still lacking, however it may be hypothesized that the increased leakiness is caused by cytokines that build up over the course of weight gain (Banks, 2005). In particular the proinflammatory adipokine lipocalin-2 may be important as this hormone is upregulated in obese humans and rodents (Wang et al., 2007), and accumulation of deamidated lipocalin-2 in arteries causes vascular inflammation and endothelial dysfunction in dietary obese mice (Song et al., 2014). While it is quite difficult to get cytokines in the CNS, they could affect penetration of other compounds. Such a possibility may exist for insulin, of which the BBB transport is increased upon lipopolysaccharide-induced inflammation (Banks, 2005). Increased BBB transport of insulin would make sense, because insulin resistance would counter its earlier mentioned neuroprotective effects. BBB endothelial cells express nuclear receptors that play a role in expression of tight junction protein, cytokine and transporter genes (Pan et al., 2011). In this respect, locally released inflammatory factors including TNFα may be a response to upregulate glucose utilization in astrocytes, turning them into a more oxidative state and actively contribute to increased neuronal vulnerability (Gavillet et al., 2008). This inflammation may then in turn causes the BBB to be weakened (Freeman et al., 2014). An increased oxidative state would lead to increased production of reactive oxygen species (ROS) and incur damage leading to a decline in cognitive function (Lu et al., 2004). Recently, Freeman et al. (2013) observed that high fat dietary-induced obese mice had significantly higher levels of total ROS, superoxide, and peroxynitrite in cortical regions compared to mice on a low fat control diet. The level of oxidative stress was highly related to the level of adiposity, and to impaired cognitive performance (Freeman et al., 2013).

## Neurodegenerative processes

The magnitude of the above mentioned derailments clearly predicts the risk for attracting dementia (Tolppanen et al., 2014), even independent of genetic and early life environmental factors (Xu et al., 2011). Thus, the collective and culminated neuronal damage that has occurred over time will inevitably lead to signs of dementia, as a function of age. Secondly, repair mechanisms may become less effective with age too (O'Neill, 2013), which then also contribute to the etiology of neurodegeneration.

### GH/IGF1

As mentioned in the introduction, the GH/IGF axis plays an important role in tissue repair and growth, and reductions in the activity of this system with age (Ho and Hoffman, 1993) may be relevant for age-related diseases. A reduction in GH secretion begins after the pubertal maximum and is correlated with an increased percentage of total body and visceral fat and decreased physical fitness (Veldhuis et al., 1997). Bartke suggested that the reduction in the GH/IGF axis is a necessary mechanism to protect against cancer (Bartke, 2008). Maintenance of the activity of the GH/IGF axis throughout life would be counter-productive in terms of hyperfunction potentially involving the mTOR pathway and this would increase damage rather than reducing it (Blagosklonny, 2013). Both dietary- and age-associated increases in adiposity are linked to a reduction in spontaneous (Vahl et al., 1997) and stimulated (Makimura et al., 2008; Cordido et al., 2010) GH secretion. This results in an overall reduction in circulating levels of GH, and these too low levels may have some repercussion for the aging process. The cause of impaired GH secretion following dietary-induced weight gain is under intense investigation (Steyn et al., 2013). A decreased pituitary GH synthesis and/or release as well as an increased GH clearance from the blood may explain the low circulating GH levels in obese rats (Dubey et al., 1988; Ahmad et al., 1989). While caloric excess results in lower GH levels, this is found to be associated with higher circulating IGF-1 and insulin levels and lower insulin sensitivity as a result of the associated weight gain (Woods et al., 2003; Sebert et al., 2005).

There is considerable evidence that GH acts in the CNS to affect cognitive processes. Humans that lack the GH receptor (GH-R) show a significant loss of spatial discrimination speed and efficiency compared to their normal relatives (Kranzler et al., 1998). This has to some extent been confirmed in animal studies (Li et al., 2011), although opposite effects also have been mentioned (Banks et al., 2010). Furthermore, GH appears to be neuroprotective against several forms of brain injuries (Hanci et al., 1994; Winkler et al., 2000; Scheepens et al., 2001; Enhamre-Brolin et al., 2013). A potential role for GH in development of the CNS is supported by the widespread distribution of GH and its receptor within the developing CNS (Garcia-Aragon et al., 1992; Lobie et al., 1993). The effects of GH in the CNS may be mediated via IGF-1, analogous to the effects seen in peripheral tissue as mentioned above. Indeed, GH or IGF1 treatment increases hippocampal neurogenesis (Aberg et al., 2000; Aberg, 2010). IGF1 (D'Ercole, 1996; Folli et al., 1996) and IGF1R (Yan et al., 2011) are expressed locally in diverse areas throughout the brain. Central administration of IGF1 decreases depressive-like behavior and brain cytokine expression, including TNFα and IL6 in mice (Park et al., 2011).

Recently it was shown that the GH stimulatory effects on cognitive function in rats with traumatic brain injury is associated with increments of hippocampal and prefrontal Brain Derived Neurotrophic Factor (BDNF) mRNA expression and its receptor TrkB mRNA expression (Zhang et al., 2014). BDNF is known to increase neurogenesis and neuronal connectivity (Mattson and Magnus, 2006), and a decline in hippocampal BDNF secretion might contribute to age-related impairment in cognitive performance (Hattiangady et al., 2005). Acutely elevating levels of IGF-1 in mice by microinjection in hippocampus was found to stimulate BNDF and cognitive performance while the opposite was observed in mice treated with IGF-1 antiserum (Trejo et al., 2007). Neuronal transport of BDNF, at least in isolated hippocampal tissue, has been shown to rely on the insulin signaling cascade including phosphorylation of PKB/Akt (Takach et al., 2015). While BDNF levels in the CNS declines with age, diet-induced obesity lowers hippocampal synaptic plasticity, BDNF levels and cognition (Stranahan et al., 2008) by itself, at least in rodents. Reduced GH and IGF1 levels, insulin resistance, as well as inflammatory markers may all contribute to this effect (Cotman, 2005).

### β-Amyloid protein

Insulin resistance and thus increased levels of circulating insulin have repercussions in the CNS. The breakdown of insulin in the CNS requires insulin-degrading enzyme (IDE), a peptide that also degrades β-amyloid protein (Aβ). Highly increased levels of insulin therefore attenuate Aβ clearance (Ho et al., 2004). Aβ is derived from a larger transmembrane amyloid precursor protein (APP) (Cras et al., 1991) of which the function is not exactly known. APP consists of a large extracellular domain, a hydrophobic transmembrane domain, and a short cytoplasmic carboxyl terminus. The Aβ sequence lies partially outside the cell membrane. A heavily glycosylated region of APP lies NH_2_-terminal to the Aβ sequence. This sequence is highly conserved and is nearly identical in species from Drosophila to human (Suh and Checler, 2002). Aβ peptides are natural products of metabolism in their monomeric form ranging from 36 to 43 amino acids (Querfurth and LaFerla, 2010), and probably serve as an antimicrobial peptides (AMPs) to stimulate the innate immune response (Soscia et al., 2010). Aβ_40_ is the most common form and is much more prevalent then the aggregation-prone Aβ_42_ species. Proteolysis of APP is required to obtain these peptides, and is facilitated by sequential enzymatic actions of beta-site APP cleaving enzyme 1 (BACE-1), a β-secretase, and γ-secretase (Querfurth and LaFerla, 2010). While being beneficial against bacterial infections (Soscia et al., 2010), Aβ can form multimers or fibrils which form the core extracellular amyloid deposits. These are surrounded and attacked by reactive astrocytes, and reactive microglia (Cras et al., 1991; Dournaud et al., 1995), probably as an adaptive response to confine infectious hazards. This process, however, can induce neuronal cell death, and will eventually result in cognitive impairment, loss of memory functions and physical deterioration, which are all irreversible (Belkhelfa et al., 2014). Correlated with fibrillization are initiation of toxic and neurotoxic cascades (Dumery et al., 2001). Aβ elicits this toxicity in its oligomeric form, and these cytotoxic properties are mediated via a ROS pathway (Dumery et al., 2001). Indeed this has also been confirmed in recent research, which stated that the formation of ROS is an early response to the oligomeric derivatives of Aβ (Giordano et al., 2014). Other studies show that amyloid plaques are colocalized with a variety of pro-inflammatory proteins and clusters of reactive astrocytes and activated microglia (Eikelenboom et al., 2010). This leads to suggest that, besides activation of the ROS pathway, a chronic inflammatory response is activated by the formation of Aβ-plaques, which may also underlie neuronal cell death and impaired cognition. Indeed, Jaeger et al. showed that mice with lipopolysaccharide-induced inflammation had increased influx and decreased steroid-independent efflux of central Aβ (Jaeger et al., 2009), which clearly would augment the etiology of neurodegenerative processes.

### Neurofibrillary tangles

Another neurodegenerative process relates to the production of neurofibrillary tangles (NFTs). These tangles are the result of a misconfiguration of microtubule stabilizing protein called tau and its associated microtubule-associated protein tau (MAPT). NFTs are made up of paired helical filaments (PHF) (Wischik et al., 1988; Dournaud et al., 1995), which can accumulate within neuronal cell bodies. This can lead to de-stabilization and eventually dissociation of microtubules. Furthermore, intermediate aggregates of tau, like Aβ, are cytotoxic and can impair cognition (Santacruz et al., 2005). This impairment might be primarily caused by synaptic failure. Indeed, subjects with mild cognitive impairment, synapses in the hippocampus begin to decline (Querfurth and LaFerla, 2010). Furthermore, the process of aging itself is also associated with synaptic loss, particularly in the dentate region of the hippocampus (Querfurth and LaFerla, 2010). As mentioned earlier, chronic inflammation, which leads to induction of senile plaques (SPs) and NFTs, is a key element in these neurodegenerative processes (Eikelenboom et al., 2010). Supporting evidence for this idea comes from a study that SPs are the source of the infection of a chronic inflammatory response that is induced by fibrillization of Aβ, which in turn activates an innate immune response (Eikelenboom et al., 2010). The Aβ peptide itself is able to induce a local inflammatory response, involving the binding of fibrillar Aβ to complement factor C1, which then activates canonical complement pathways in an antibody-independent fashion (Eikelenboom et al., 2006). This activation can play an important role in the migration and activation of microglial cells which express complement factors. These microglial cells in turn show a high secretion level of IL6 and TNFα (Familian et al., 2006; Eikelenboom et al., 2010) in the case of high concentration of Aβ.

In type-2 diabetic patients, the production of Aβ is exacerbated (Sims-Robinson et al., 2010). Furthermore, studies in mice have revealed that in both type 1 and particularly type 2 diabetes the phosphorylation of tau is increased (Kim et al., 2009). In this case the consideration must also be made about the fact that cognitive deficits might not only be caused by tau phosphorylation, but also by toxicity of hyperglycemia, or by impairment of insulin signaling *per sé*. Specifically, glycosylation of O-linked β-*N*-acetylglucosamine (GlcNAcylation) underlies the etiology of glucose toxicity in diabetes mellitus, and also plays a role in neurodegenerative processes. The decreased level of GlcNAcylation under diabetic conditions therefore suggests an attenuating role on tau phosphorylation and tau-induced neuronal death (Liu et al., 2009). Patients with type-1 diabetes mellitus exhibit most problems with cognition because of a systemic insulin deficiency. Changes in spatial learning and hippocampal long-term potentiation (LTP) are indeed seen in animal models of diabetes mellitus (Sims-Robinson et al., 2010). These changes can be normalized or prevented by insulin replacement therapy. Within the case of type-2 diabetes mellitus cognitive impairment and neurodegeneration could be caused by a loss of sensitivity of insulin receptors which might lead to an increased expression of Aβ and tau (Sims-Robinson et al., 2010). Furthermore, a decrease in insulin receptor signaling leads to inhibition of PKB/Akt, which normally dephosphorylates (i.e., activates) glycogen synthase kinase 3β (GSK-3β), and that finally leads to tau hyperphosphorylation (Sims-Robinson et al., 2010). Activation of GSK-3β is also highly relevant to TNFα, since TNFα can inactivate PKB/Akt via TNRF1, which leads to inactivation of GSK-3β. Finally, down-regulation of GSK-3β leads to apoptosis (Takada et al., 2004). Collectively, this leads to the suggestion that there is a connection between insulin resistance and a potential neuroinflammatory response via up-regulation of TNFα.

### Balancing TNFα

Important for consideration is the fact that the binding and signal transduction of TNFα is mediated by two TNF receptors; i.e., TNF-Receptor 1 (TNFR1) and TNF-Receptor 2 (TNFR2) (Zettlitz et al., 2010). TNFR1 excerpts axonal and neuronal damage through its pro-inflammatory effects, which are clearly observed under chronic inflammatory situations (Fischer et al., 2011). TNFR2, however, is associated with a neuroprotective effect against excitotoxic insults, although this has to be confirmed *in vitro* (Marchetti et al., 2004). TNFR2 is considered as an activator of the NF-κB pathway, which exerts gene activation and anti-apoptotic signaling, but this also has a pro-inflammatory response via TNFR1 (Kontermann et al., 2008). TNFR1 contains a death-domain (DD), which for that reason can mediate apoptotic signals, while TNFR2 can mediate cell survival and neuronal protection via the NF-κB pathway (Wajant et al., 2003). This crosstalk is mostly mediated via the TNF-receptor associated factor (TRAF) family, with TNFR1 activation gene expression via indirect recruitment of TRAF1, and TNFR2 recruiting TRAF2. These factors form the basis for TNF cytokine signaling (Inoue et al., 2000). Thus, there is a dual role of TNFα on neuronal homeostasis, which is mediated via these two receptors.

The finding that TNFα is not solely produced by macrophages and other immune-regulating cells, but also by adipose tissue in obese (and insulin-resistant) mice (Moller, 2000) and even in human adipocytes in individuals with obesity or hyperinsulinemia, may suggest a causal link between processes in the brain and the periphery. Weight loss in obese individuals correlates with a reduction of circulating TNFα levels and other inflammatory cytokines (Bruun et al., 2003), which may eventually lead to a reduced activity of neuroinflammatory pathways in the brain (Ghanim et al., 2012). Thus, this evidence collectively suggests that obesity and/or diabetes mellitus induces a neuroinflammatory response which upregulates TNFα and other pro-inflammatory cytokines that via multiple pathways leads to an increased risk of neurodegeneration.

## Progression toward Alzheimer's disease

The processes and pathways mentioned above can at some point lead to such cognitive impairments that they become classified as AD. Memory loss is the most presenting symptom found in people developing AD (Burns and Iliffe, 2009). Also present are symptoms as poor sleep quality (Landry and Liu-Ambrose, 2014), emotional changes, anxiety and depression (Burns and Iliffe, 2009). This cognitive deterioration furthermore coincides with the neuropathological staging of AD (Braak and Braak, 1991; Schroeter et al., 2009). At the age AD manifests itself (frequently around the age of 65) (Brookmeyer et al., 1998), the neurodegenerative processes are already ongoing for many years, or perhaps even decades, yet its progression can differ substantially between patients. Advancing dementia in AD correlates strongly with the disproportional loss of synapses between neurons (DeKosky and Scheff, 1990). The number of NFTs is directly related to the severity of AD, and could serve as a pathologic marker (Querfurth and LaFerla, 2010). The initial indication that led to show the contribution of TNFα in AD was the presence of TNFα in the vicinity of SPs in post-mortem AD brains (McCoy and Tansey, 2008). This co-localization of TNFα and SP was also seen in brains of transgenic mice that expressed APP. The fact that transgenic mice with deletion of TNFR1 show a significant decline in microglia activation, BACE1 activity, Aβ pathology, less memory deficits and less neuron loss indeed suggest a role for TNRF1 in the pathogenesis of AD (He et al., 2007). A potent and effective method of treating AD via the TNF signaling pathway is therefore anticipated. Targeting the TNF signaling pathway in neurodegenerative diseases such as AD is a strategy, which has received increasing amounts of interest. Human-specific reagents, which target the neurodegenerative and neuroprotective effects of respectively TNFR1 and TNFR2 are for example ATROSAB, a human specific antagonistic TNFR1 antibody (Zettlitz et al., 2010), and Tenascin C single chain TNF (TNC-scTNF) (Fischer et al., 2011), a TNFR2 selective agonist. By selectively inhibiting TNFR1 and/or activating TNFR2, the neuroprotective effects of these receptors might be of use as a possible break on the neuroinflammatory pathway involved in neurodegenerative processes.

Several studies have already confirmed that patients with diabetes mellitus have an increased risk of developing AD (Brands et al., 2005; Biessels et al., 2006). About 80% of patients with AD exhibit symptoms of glucose intolerance or diabetes mellitus (Janson et al., 2004). Several factors associated with obesity including hyperglycemia, insulin resistance, glucose intolerance, atherosclerosis, adiposity and hypertension (Haan, 2006) can increase the risk for attracting AD too. Interestingly, being overweight or obese more strongly increases the risk for developing AD later in life, than being overweight or obese at late life (Emmerzaal et al., 2015). In their meta-analysis on epidemiological studies on the link between obesity and AD, Emmerzaal et al concluded that “current trends of more overweight and obesity in childhood and adolescence may translate to longer exposure of the brain to potentially detrimental vascular and metabolic effects of adipose tissue. This increased size of the population at risk in combination with longer survival, continues to underscore the importance and necessity of research in this field” (Emmerzaal et al., 2015). In the following paragraphs, a number of factors are described which have been mentioned to affect the risk for attracting AD.

### ApoE

Early-onset familial AD (EOAD) (under 65 years of age) is usually the cause of an age-related dichotomy of rare autosomal dominant mutations (Bertram et al., 2007). The risk of late-onset AD (LOAD) (over 65 years of age) is on the other hand increased by genetic variants of which the penetrance is relatively low, but prevalence high (Tanzi, 1999). LOAD probably accounts for about 95% of all AD cases. The one genetic factor that has been firmly established to underlie LOAD is the ε 4 allele of Apolipoprotein E (ApoE, ApoE-ε 4). ApoE is a protein that is mainly involved in the transport of cholesterol (Strittmatter et al., 1993). The production of ApoE is mediated by astrocytes, which then secretes it into the CNS in high-density lipoprotein (HDL)-like particles. ApoE levels are upregulated following injury and during development, so it functions mainly in growth and repair (Strittmatter et al., 1993). An increase of ApoE is also seen in several chronic neurodegenerative diseases, such as AD, in which ApoE binds to SP and NFT (for review see Liu et al., 2013). The suggestion that ApoE serves as a genetic predisposing factor comes from the observation of high levels of ApoE mRNA in brains of patients with AD (Strittmatter et al., 1993). Through the action of the ATP-binding cassette transporter (ABCA1) and other related transporters, ApoE is lipidated. ABCA1 normally serves to transfer phospholipids and cholesterol to ApoE (Jiang et al., 2008). Research suggests that the lipidated form of ApoE acts to enhance the clearance of Aβ peptides from the brain, in the form of elevated proteolytic breakdown. There are multiple isoforms of ApoE, of which ApoE-ε 4 is one of these. The ApoE-ε 4 isoform differs structurally from other isoforms resulting in the fact that this ApoE-ε 4 protein mediates neuronal death and neurodegeneration, and thus not have the same Aβ proteolytic properties (Bagyinszky et al., 2014).

As opposed to LOAD, EOAD is characterized by mutations in the APP gene, presenilin 1 (PSEN1) gene and presenilin 2 (PSEN2) gene. Mutations in these genes might result in alteration of Aβ production (both Aβ_40_ and Aβ_42_), which can lead to apoptosis and dementia (Bagyinszky et al., 2014). The involvement of the APP gene has been discovered in patients with Down syndrome who are prone to develop AD at a very early age. Triplication of the APP gene, which is localized on chromosome 21, is the cause of this greater predisposition of early onset of AD pathology in patients with Down syndrome (Bagyinszky et al., 2014).

Because of the involvement of ApoE in lipid-transport and its role in the clearance of Aβ, this is a significant risk factor to discuss. ABCA1 expression, because of its lipidation action of ApoE, is a crucial transporter-protein. The expression of both ABCA1 and ApoE are induced by the Liver X receptor (LXR), which is a ligand-activated transcription factor (Jiang et al., 2008). LXR acts as a sensor for cholesterol and is activated by oxysterol (oxidized derivatives of cholesterol), which results in a rapid increase of lipidated forms of ApoE (Cao et al., 2007; Jiang et al., 2008). This is where lifestyle factors first come into play in the onset and progression of AD, because it suggests a strong link between AD and dietary habits, the cardiovascular system, and metabolism. For example, it has been shown that high systolic blood pressure at midlife is a significant risk for developing AD later in life (Kivipelto et al., 2001). Furthermore, the same research also revealed a link between high serum cholesterol concentrations as a risk for AD. Combining these two risk factors probably contribute in a synergistically fashion to the proneness of AD. The mechanism behind these interactions however remains quite complex, and clearly more research is necessary to unravel them. It may however be speculated that a high cholesterol intake elevates the serum cholesterol levels, which create an overload of ApoE, which then leads to a down-regulated proteolysis of Aβ (Puglielli et al., 2003). Susceptibility for AD increases further in carriers of the ApoE-ε 4 SNP, because of the higher cholesterol content of the ApoE-ε 4 lipoproteins. Furthermore, in patients with AD, lecithin cholesterol acyltransferase (LCAT) activity is significantly reduced, suggestively by the ApoE-ε 4 SNP (Puglielli et al., 2003). LCAT is an enzyme that catalyzes the process that clears cholesterol from peripheral cells.

TNFα may interact with ApoE, at least in atherogenic processes in mice that were feeding a diet rich in saturated fat and cholesterol (Ohta et al., 2005). This upregulation of TNFα is probably involved in the inflammatory responses in atherogenesis, mediated mainly by its soluble form of the TNFR1. Whether this TNFR1 mediated pathway provides the link between a high fat/cholesterol diet and the neuroinflammatory responses, which can promote the onset of AD remains to be investigated.

### Saturated dietary fat

As mentioned earlier, a diet with a high level of saturated or mono-unsaturated dietary fat contributes far stronger to abdominal weight than a diet with a poly-unsaturated fat content (Jang et al., 2003). The statement that a high fat diet may be a contributing factor in neuroinflammation is partially established by the fact that a high fat diet has been reported to induce a CNS inflammatory response by means of an enhanced systemic inflammation (Timmermans et al., 2014). Furthermore, numerous previous studies have shown the effect of high fat feeding on microglial activation and overall neuroinflammation in specific brain regions (Thaler and Schwartz, 2010; Fuente-Martin et al., 2012; Thaler et al., 2012). In accordance, considering the risk factors age and obesity, which are both associated with neuroinflammation (Labrousse et al., 2012; Makki et al., 2013), also seem to both have a lowering on overall testosterone levels in males (Teerds et al., 2011; Jayaraman et al., 2014) which then in turn also affects neuroinflammation in rodents (Jayaraman et al., 2014). Particularly high levels of saturated fatty acids (SFA) may increase the risk of dementia and even AD later in life (Kalmijn et al., 1997; Morris et al., 2004; Eskelinen et al., 2008), suggestively via mechanisms involving ApoE-e4 (Laitinen et al., 2006) and may also be conveyed by inflammatory mechanisms (James et al., 2000). The same study suggests the counteracting effect of omega-3 poly-unsaturated fatty acid (n3-PUFA) ingestion on systemic inflammatory cytokines such as TNFα (James et al., 2000). Recent studies concur with this suggestion, showing that specific n3-PUFAs exert profound anti-inflammatory effects on the brain after neuroinflammatory challenges (Bazan, 2007; Belayev et al., 2009; Orr et al., 2013; Zendedel et al., 2015). Particularly decreased levels of the n3-PUFA docosahexaenoic acid (DHA) correlate with cognitive impairment and are established to be decreased in AD (Prasad et al., 1998; Suzuki et al., 1998; Bazan, 2007; Orr et al., 2013). It is suggested that Western diets do not provide the aged brain with an optimal supply of these n-3 PUFAs (Woo, 2011; Cutuli et al., 2014). Even more convincing are the facts that the aged brain shows a reduced capacity of n-3 PUFAs to pass the BBB and to convert shorter chained fatty acids to longer fatty acids (Yehuda, 2012; Cutuli et al., 2014). There is a possible beneficial effect of n-3 PUFA supplementation (including DHA) that may improve hippocampal functioning in aged mice (Cutuli et al., 2014). Unfortunately, the aforementioned ApoE-e4 carriers appear to be unresponsive to the protective effect of n3-PUFA supplementation (Barberger-Gateau et al., 2011). Furthermore, n-3 PUFA supplementation seems inefficient at old age to prevent cognitive decline or when AD has been diagnosed already, and should thus be regarded as preventive rather than curative (Cederholm et al., 2013; Jiao et al., 2014)

### Sedentary lifestyle

Leading a sedentary life style is known to be a factor that contributes to weight gain, an at a population level a reduction in obesity and sedentary living increases the number of years without cognitive impairment (Anstey et al., 2014). Chronic exercise is also associated with preservation of overall cognitive function and prevention of dementia (Zhao et al., 2014). It is difficult to dissociate the beneficial effects of physical activity on cognitive function independent of its effects on peripheral endocrine and cardiometabolic health. However, exercise is a proven stimulus of GH release and an acute bout of exercise stimulates a significant GH pulse (Pritzlaff et al., 2000; Wideman et al., 2002; Godfrey et al., 2003), although the effects in the elderly seems diminished (Marcell et al., 1999). However, resistance training over a 12 week exercise protocol in elderly people promoted an increase in acute GH response, possibly owing to an increase ability to exert oneself (Craig et al., 1989). Thus, exercise is a potent stimulator of GH and IGF1 release and it may take a few hours to recover back to baseline levels (Kanaley, 2008). It is speculated that the mechanism responsible for exercise-induced GH release is a suppression of hypothalamic secretion of somatostatin and possibly during-high intensity exercise an augmented hypothalamic secretion of GHRH. The primary function of GH release during exercise may be post exercise protein synthesis (Kanaley, 2008). Basal levels of IGF-1 decrease during aging (Yamamoto et al., 1991; Ruiz-Torres and Soares de Melo Kirzner, 2002; Trejo et al., 2004). Trejo et al. (2002) have pointed out that physical activity increases the uptake of IGF into CNS, and IGF may activate the synthesis and release of nerve growth factors (NFGs). In rodent studies, exercise is known to stimulate BDNF expression and release in the hippocampus, in this is associated with increased cognition and increased PKB/Akt phosphorylation (Aguiar et al., 2011). In addition, wheel-running aged mice had attenuated microglial proliferation and increased expression of a proneurogenic hippocampal phenotype compared to sedentary mice (Kohman et al., 2012). Stranahan et al. observed that exercising mice had increased hippocampal BDNF levels and associated increased spine density (i.e., compared to sedentary mice), and this effect was also observed in genetically obese mice, yet less outspoken (Stranahan et al., 2008). Interestingly, elevating hippocampal BDNF signaling by the TrkB receptor increases “browning” of white adipose tissue and adaptive thermogenesis (Cao et al., 2011), which would constitute a positive feedback, and may explain the positive effects of physical activity on metabolic as well as cognitive health.

Finally, long-term exercise treatment reduces oxidative stress in the hippocampus of aging rats (Marosi et al., 2012). Furthermore, it has also been shown in a mouse model that exercise can attenuate the DNA binding of NF-κB, which essentially suggests a decrease in inflammation, possibly involving TNFα. Healthy volunteers who received an injection with a low dose of *Escherichia coli* endotoxin showed a two- to threefold increase of circulating TNFα, and this was significantly reduced by exercise (Petersen and Pedersen, 2005). Because ROS and inflammatory cytokines were discussed earlier to be involved in an early response to the oligomeric derivatives of Aβ, exercise would therefore slow progression of AD. Exercise can also increase vagal tone, which suggestively reduces the inflammatory response because of the aforementioned cholinergic anti-inflammatory response (Woods et al., 2012). In summary, exercise affects multiple organ and organ systems, which contribute via different mechanism to delay the onset and progression of AD.

### Stress

It was mentioned earlier that obesity is associated with an increase in sympathetic activity (Arrone et al., 1997; Skrapari et al., 2007; de Jonge et al., 2010) and HPA axis activity (Bjorntorp, 1991; Benthem et al., 2000), which both could result from a reflex mechanisms involving fatty acid spill-over from abdominal fat depots toward the liver. Psychological or psychosocial stress also lead to enhancement of sympathetic activity and HPA axis activity, which are required to fuel a fight/flight response (Koolhaas et al., 2011). In our society of plenty, however, fight/flight responses are no solution for our stressors to which we are exposed. Furthermore, living in a complex society can impose stressors that are chronic in nature. As a result, stress responses become extended, and without the physical component of stress would incur damage (McEwen and Wingfield, 2003), with the higher spill-over of fat and glucose propagating sympathetic and HPA axis activity. Eating highly palatable food items can be rewarding and may be protective against deterioration of mood (Dallman, 2010), but would not fuel physical activity, and thus would enhance cardiometabolic damage. Reader et al used a repeated social defeat (RSD) stress model for induction of chronic psychological/psychosocial stress and observed increased microglial activation, which is a proxy for neuroinflammation (Reader et al., 2015). In a psychosocial stress model, submissive animals develop depressive-like behavior (Norman et al., 2010), which is often associated with an increased inflammatory state (Cohen et al., 2012). The mechanistic links between inflammation and depression are well understood (Norman et al., 2010; Capuron and Miller, 2011; Reader et al., 2015), and may involve HPA axis activity and sympathetic activity (Reader et al., 2015). A permissive action of the endothelial IL1 receptor and stress-induced neuroinflammation and anxiety-like behavior was recently established (Wohleb et al., 2014a), and monocyte trafficking across the BBB probably plays an important role in this link (Wohleb et al., 2014b).

Increasing parasympathetic activity might have beneficial effects on inflammation, as reviewed by Tracey (2007). As already mention, Tracey exposed the effects of stimulating the vagus nerve and administrating nicotine which inhibits TNFα release and various other pro-inflammatory cytokines (Borovikova et al., 2000; Wang et al., 2003; Tracey, 2007; Pavlov and Tracey, 2012). Increasing parasympathetic activity would also normalize autonomic disbalance in the obese state, in which sympathetic activity is known to be increased (Arrone et al., 1997; Vaz et al., 1997; Grassi et al., 2005; Skrapari et al., 2007; de Jonge et al., 2010). Thus, by increasing parasympathetic tone, the vicious cycle including increased fatty acid spill-over and increased sympathetic and HPA activity would be broken. Indeed, vagal nerve stimulation (VNS) has been used in the treatment of AD. Vonck et al. (2014) recently gave an overview of the studies addressing VNS, and alluded to the idea that anti-inflammatory pathways in the CNS are set in motion by VNS that are beneficial for AD patients. Whether this would also work under conditions of psychosocial or psychological stress is, to our knowledge, unclear. Because stress augments Aβ release and amyloid aggregation in animal models of AD (Dong and Csernansky, 2009; Srivareerat et al., 2009) further research in this field is therefore warranted.

### Circadian disorders

Finally, the role of circadian dysregulation as a considerable risk factor for neuroinflammatory responses deserves some attention, as it will increase the risk of neurodegenerative diseases. Circadian disorders are primarily caused by deficits in the circadian system driving the 24 h rhythms in body and brain. In the brain, the hypothalamic suprachiasmatic nucleus (SCN) has been identified as the master circadian clock driving many other circadian clocks and clock systems. One important function of this clock is the regulation of the sleep/wake cycle. Disruptions of the circadian clock will lead to sleep disturbances, and these sleep disturbances (e.g., sleep loss) in turn cause a neuroinflammatory response (Wisor et al., 2011). As such, circadian disorders can be viewed as a distinctive pro-inflammatory factor. It has been recently recognized that improvements in circadian rhythm functioning as well as appropriate sleep interventions should therefore be further explored in order to delay neurodegenerative progression, and notably so in case of AD (Landry and Liu-Ambrose, 2014). The age-related alterations in innate immunity and chronic systemic inflammation cause a persistent neuroinflammatory state. This state accelerates degenerative diseases via its dysregulation of clearance mechanisms of misfolded or damaged neuronal proteins and loss of axonal integrity linked to tau-pathology (Krstic and Knuesel, 2013). In a study comparing the SCN of young and aged mice, it was found that neurons and glial cells of the SCN are sensitive to inflammatory signals (Sadki et al., 2007). The response of the aged glial cells in the SCN is markedly more pronounced to cytokine exposure (Bentivoglio et al., 2006). In the aged SCN, it is notably the circadian output toward other hypothalamic nuclei that is disrupted (Van der Zee et al., 1999; Weinert, 2000). A successful intervention would be the one, which improves circadian output pathways. One such intervention could be exercise, as it has been shown to strengthen the circadian system in aged mice (Leise et al., 2013). The cholinergic system provides input to the circadian clock, and it has been hypothesized that reduced cholinergic input hampers several circadian clock-dependent processes, including sleep as well as learning and memory (Hut and Van der Zee, 2011; Mulder et al., 2013). As acetylcholine release inhibits the production of proinflammatory cytokines by microglia (Shytle et al., 2004), the cholinergic system has been suggested to function as a mediator in neuroinflammatory responses. However, neuroinflammation has been shown to selectively disrupt the cholinergic system (Araujo et al., 1989). Hence, increasing levels of neuroinflammation impair the cholinergic system and reduce its inhibitory role on proinflammatory cytokine production, further worsening the situation. Taken together, the aging SCN is at increasing risk of becoming damaged by (excessive) neuroinflammation. This will lead to (further) circadian dysregulation, and as a result increased disturbance of the sleep/wake cycle and hence increasing excessive neuroinflammatory responses even further. Obesity is known to be associated with circadian (Roenneberg et al., 2012) and sleep (Kudlow et al., 2013) derangements, and circadian disturbances inflate the metabolic syndrome (Staels, 2006). Improving the function of the SCN and maintaining the expression of circadian rhythmicity should therefore be one of the critical interventions toward slowing down neurodegeneration caused by neuroinflammatory processes.

## Concluding remarks

Neuroinflammation, processing of APP to the Aβ peptide, tau protein hyperphosphorylation, relocalization and deposition, are the leading mechanisms in AD. These processes are propagated by obesity, the metabolic syndrome and type-2 diabetes mellitus. Stress, sedentariness, dietary overconsumption of saturated fat and refined sugar, and circadian derangements/disturbed sleep all contribute to obesity and related metabolic diseases, but also accelerate age-related damage and senescence that all feed the risk of developing AD. It is difficult to make clear separations between these mechanisms, as they are strongly overlapping and integrated.

The choice for addressing above-mentioned issues in relation to the TNF system was provided by its major role in immune regulation with widespread implications. TNFα is however not the least complex in this case. Although many mechanisms have been mentioned with respect to TNFα, these are probably the “tip of the iceberg.” This is for example illustrated by the complex crosstalk that exists between the two TNF-receptors: TNFR1 and TNFR2. TNFR1 translates mainly proinflammatory signals, while TNFR2 is thought to primarily be a pro-survival signal (Naude et al., 2011). Because of the diversity of functions of TNFα, which is not restricted to either TNFR1 or TNFR2, it becomes very complex and can be dependent on the temporal dynamics of this crosstalk and under the given (patho) physiological condition in which they take place.

**Figure 1:**
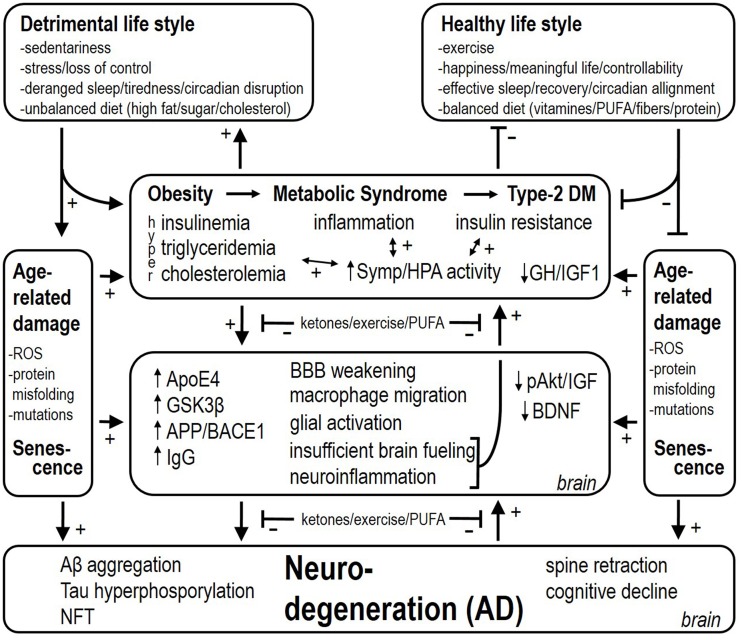
**Detrimental and healthy life style factors respectively stimulate and reduce the risk for attracting obesity, the metabolic syndrome and type-2 diabetes mellitus**. These factors also have a stimulatory effect *per sé* on age-related damage and accelerate senescence. Associated with obesity, metabolic syndrome, and type-2 diabetes mellitus, there is inflammation, insulin resistance, and hyperinsulinemia (i.e., depending on the stage of type-2 diabetes mellitus). Hyperactivity of the sympathetic nervous system and HPA axis worsen insulin resistance and deteriorate cardiovascular health. At the level of the brain, several mechanisms are set in place, among which neuroinflammation and insufficient brain fueling are proposed to backfire and contribute to increased sympathetic and HPA activity as well. Finally, these processes lead up to Aβ aggregation, Tau hyperphosphorylation, and NeuroFibrillary Tangle (NFT) deposition, which also backfire and culminate into AD. (1) Upregulating ketone provision to the brain, (2) exercise/physical activity, and (3) increasing the level of n-3 PUFAs (by diet supplementation as preventive action) slow the progressive stages in the disease, and are also proposed to reduce the backfiring effects, which puts a brake on the vicious cycles in the etiology of AD.

As to why AD develops, we speculate that this is initially an adaptive response of the brain that becomes maladaptive. Inflammation in the brain, analogous to inflammation in peripheral organs and tissues, is a response to damage and requires fuels for restorative actions. Migration of macrophages and monocytes across the BBB and glial activation are highly adaptive in this sense too. Weakening of the BBB endothelium in the obese state allows this migration to occur. The brain requires glucose for metabolic purposes, however insulin resistance in certain brain regions makes this a problematic event. The deficient energy status implicated in the neurodegenerative pathology has previously been coined “type-3 diabetes mellitus” (Steen et al., 2005). Thus, a coordinated response of a chronically injured brain (which we believe occurs with obesity and particularly with metabolic diseases like type-2 diabetes mellitus) would be to increase fuel availability by (1) activating sympathetic nervous system outflow and HPA axis activity, and (2) promote food consumption. Increased basal and cold-induced sympathetic and HPA axis activity have indeed been demonstrated in AD patients (Pascualy et al., 2000). In the case of obesity and metabolic derangements, these responses would obviously worsen the etiology of the disease. Besides glucose, the brain can also utilize ketones for metabolic purposes (Owen et al., 1967). Ketone bodies are elevated in conditions of food restriction, and both food restriction as well as ketone body supplementation are neuroprotective, but hypoglycaemia (another feature of food restriction) is not neuroprotective (Davis et al., 2008). Feeding a diet very low in carbohydrates and usually rich in proteins and fat is also ketogenic, and has been shown to slow the progression of AD too (Gasior et al., 2006). The neuroprotective effects may result from enhancing energy provision by ketones to resist or fight metabolic and inflammatory challenges (Julio-Amilpas et al., 2015). We propose here that the brain misinterprets “obesity” for “starvation.” Thus, both obesity and starvation are characterized –among others–by shutting down leptin access toward the brain, insulin resistance, and hypertriglyceridemia, but the compound to fuel neuronal metabolic processes for repair (i.e., ketones) is lacking in obesity. For this reason obesity and associated metabolic disease may underlie an energy crisis in the brain that leads to an insufficient counteraction of accumulating damage and finally will increase the risk to develop AD (see Figure 1).

Sustainable health is important for one's physical and mental well-being, social presence and quality of life, apart from the absence of disease (World Health Organization, 2015a,b), but this is in first place achieved by making healthy and conscious lifestyle decisions in which ample exercise and sleep, healthy dietary habits, and a manageable mental stress level are defining factors. Eventually, these choices can be traced to a molecular level and very specifically translated into how and where these have an action on, but also were they can go awry and eventually develop into disease. Once a disease -like AD- has developed, the underlying mechanisms are often obscured. Only studying the adaptive and maladaptive processes from the earliest possible stage onwards at the highest level of molecular and temporal resolution without forgetting the bigger picture in both humans as well as animal models will eventually unravel the exact underpinnings of AD.

### Conflict of interest statement

The authors declare that the research was conducted in the absence of any commercial or financial relationships that could be construed as a potential conflict of interest.
